# P67 PIMS-TS MDT – where do we go from here?

**DOI:** 10.1093/rap/rkac067.067

**Published:** 2022-09-28

**Authors:** Hanna Lythgoe, Alicia Pakenham, Janet McDonagh, Emily Willis, Phil Riley

**Affiliations:** Royal Manchester Children's Hospital, Manchester, United Kingdom; Royal Manchester Children's Hospital, Manchester, United Kingdom; Royal Manchester Children's Hospital, Manchester, United Kingdom; Royal Manchester Children's Hospital, Manchester, United Kingdom; Royal Manchester Children's Hospital, Manchester, United Kingdom

## Abstract

**Introduction/Background:**

Following first reports of paediatric inflammatory multisystem syndrome temporally associated with COVID-19 (PIMS-TS) in April 2020, services have rapidly been developed to manage these patients. In our tertiary paediatric rheumatology service a daily virtual PIMS-TS multidisciplinary (MDT) team meeting was set up in January 2021. This meeting facilitates discussions between the tertiary centre (routinely including paediatric rheumatology and infectious diseases/immunology teams) and general paediatric teams in district general hospitals (DGHs) and within our centre. The aim of this project was to evaluate the service and understand general paediatric opinion in order to consider the future direction of the meeting.

**Description/Method:**

We looked at a one month period after meetings were initiated and compared it with a one month period a year later (February 2021 and February 2022) to define patient numbers and outcomes. Alongside this we constructed an online survey aimed at general paediatricians to determine opinion of the current structure of the MDT and how it may be developed in the future. The survey was sent to general paediatricians within our own centre and in the eleven DGHs falling within our region. Results were analysed descriptively.

**Discussion/Results:**

During February 2021, 19 new referrals were discussed within the PIMS-TS MDT; each referral was discussed for a median of 5 days (interquartile range (IQR) 3–6 days). Of these, 11/19 (58%) had a final primary diagnosis of PIMS-TS and 5/19 (26%) patients were transferred for tertiary care (of whom 4/5 (80%) had PIMS-TS). In February 2022, 14 new referrals were discussed for a median of 2.5 days (IQR 2–5.75 days). Of these, 3/14 (21%) had a final diagnosis of PIMS-TS and 2/14 (14%) were transferred for tertiary care (of whom neither had PIMS-TS).

We received responses from 20 general paediatricians covering 9/11 (82%) DGHs within our region plus our own centre. Most clinicians had discussed up to 6 patients in the meeting (9/20 (45%) 1-3 patients; 9/20 (45%) 4-6 patients; 2/20 (10%) >6 patients). All clinicians felt the MDT helped facilitate appropriate diagnostic work-up and treatment decisions. 18/20 (90%) felt that the meeting helped avoid unnecessary tertiary paediatric transfers. Interestingly, 9/20 (45%) felt that a routine PIMS-TS MDT meant they were more likely to discuss a patient with rheumatology (1/20 (5%) less likely). All clinicians felt the meeting improved care for patients and most felt it increased their confidence in looking after patients with PIMS-TS (19/20, 95%) and was useful for continuing professional development/training experience (19/20, 95%).

Considering the future direction of the meeting, all clinicians felt it should be continued but most (16/20, 80%) felt it should be aimed at a wider patient group. 11/20 (55%) felt a later time of day would be more convenient (currently 11am). Over half (11/20, 55%) thought it should be combined with a currently separate meeting for acute COVID-19 patients (7/20 (35%) don’t know; 2/20 (10%) no). A minority (4/20, 20%) reported difficulty accessing the meeting.

**Key learning points/Conclusion:**

As the initial phase of the pandemic draws to a close and numbers of PIMS-TS cases decline this is important data to reflect on how services can go forward into the next phase. While numbers of PIMS-TS cases reduced, the meeting was still well-used and evolved to include patients with other diagnoses. The survey confirms that most general paediatricians believe it improves patient care and would like the meeting to continue but that review of the format may be helpful. Particular considerations are to broaden the scope of the meeting beyond PIMS-TS, revise the timing and consider how to improve ease of access to the meeting for all.

Further work will focus on evaluating patient numbers and diagnoses over the full period of the PIMS-TS MDT and adapting the format of the current MDT in response to the feedback received.

